# Raising the temperature: A critical geographical perspective on heat

**DOI:** 10.1177/27539687251331070

**Published:** 2025-05-19

**Authors:** Caitlin Robinson

**Affiliations:** School of Geographical Sciences, 1980 University of Bristol, Bristol, UK

**Keywords:** Heat stress, climate extremes, vulnerability, thermal inequalities, heatwaves

## Abstract

Heat is an increasingly defining characteristic of life in diverse regions globally, contributing to more human mortality than any other climate-related weather. As a spatio-temporal phenomena that is at once physical and meteorological, as well as environmental, social, technical, cultural, embodied, and political, geographers have much to contribute towards understanding heat and its differential impacts. However, critical geographical research on heat is relatively disparate. This paper reviews existing perspectives encompassing inequalities and vulnerabilities; governance and violence; infrastructure and labor; cultures and practices; and atmospheres and attunements. We argue that critical (human) geography should foreground heat, and its complex materiality.

## Introduction: Heat in the twenty-first century

Strange weather brings out strange behavior. As a Bunsen burner applied to a crucible will bring about an exchange of electrons, the division of some compounds and the unification of others, so a heatwave will act upon people. It lays them bare, it wears down their guard. They start behaving not unusually but unguardedly. They act not so much out of character but deep within it. (Instructions for a Heatwave, O’Farrell 2013)

**Figure 1.**
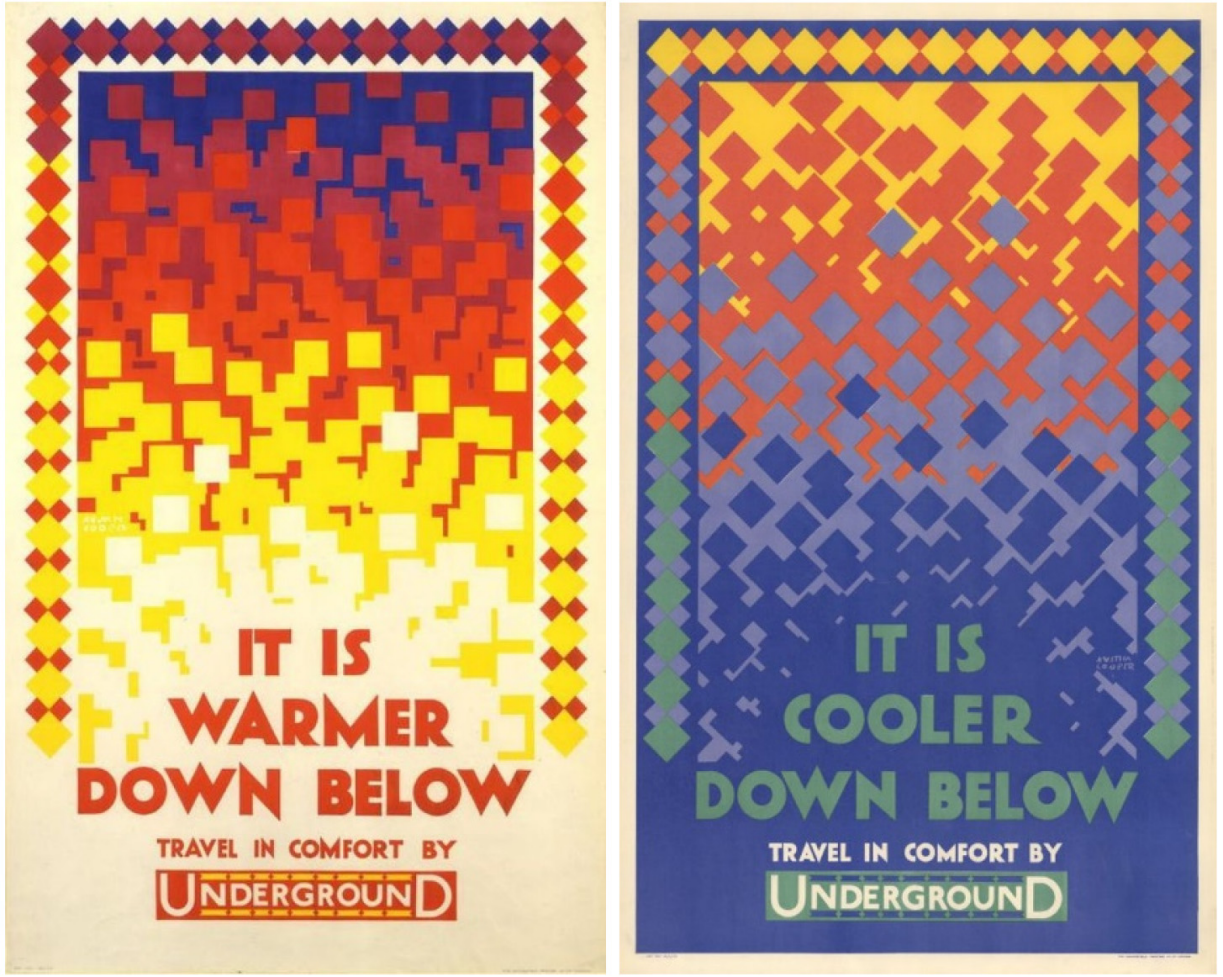
London Underground posters: It is warmer down below (left) and It is cooler down below (right). Figures are licensed ©TfL from the London Transport Museum collection.

Advertisements on the London Underground have long used ambient dimensions to sell the benefits of travel. Since the 1920s passengers have been no stranger to posters proclaiming to transport them to parks for “fun and fresh air,” or the “exceptionally healthy” suburbs of the United Kingdom's (UK) capital city. These adverts also extended to (un)comfortable temperatures. In 1924, Austin Cooper was commissioned to create two posters to advertise the benefits of travelling underground—a winter iteration declaring “it is warmer down below,” and a summer version claiming “it is cooler down below”—tapping into passenger needs for comfortable temperatures when planning their journey (Figure 1). Fast forward to 2023 and much has changed. The underground is dealing with excessive heat due to deep and poorly ventilated tunnels. Contending with a changing climate and urban growth, rather than being a mechanism for finding comfort from urban heat, the underground exposes passengers to dangerously high temperatures during the summer^1^.

Heat is a defining characteristic of the twenty-first century given a changing climate, rapid urbanization, and a growing tendency towards atmospheric control (Starosielski 2021). Heat stress contributes to more human mortality than any other climate-related extreme weather globally (WMO 2023). A growing urban population is at risk of extreme heat, with global exposure increasing by 200% between 1983 and 2016, affecting 1.7 billion people (Tuholske et al. 2021). Mass die-offs of animals, plants, and microorganisms have been recorded in response to heat stress—from songbirds to tropical forests—the sharp end of wider shifts by populations to cope with temperature change (Locosselli et al. 2020; Sanderfoot et al. 2022).

Designed by Cooper (1924). Reproduced by London Transport Museum (2021).

Whilst heat exchanges are universal, occurring between all sorts of collectives of bodies and objects, they are by no means uniform. As climate justice debates highlight, typically, those most exposed to heat stress are least responsible for changes in temperature, or the configuration of urban environments (Sultana 2022). Thermal inequalities are therefore increasingly central to understanding life in the Anthropocene for diverse, often marginalized, populations.

In 2024 alone, the city of Delhi, India, experienced a prolonged heatwave over many weeks with daily temperatures peaking at almost 50 °C. Exacerbated by intense humidity, water shortages, and power outages due to record breaking demand for energy for air conditioning, these challenges were particularly acute for the third of the population that live in congested and poor-quality informal housing, which often lacks adequate cooling facilities, and for precarious populations working outdoors. Meanwhile in June of the same year, in Saudi Arabia, 1,300 Hajj pilgrims died from exposure as they walked long distances in direct sunlight with insufficient shelter, exposed to temperatures that climbed to 51.8 °C. In this warming age, there are growing calls for greater accountability for mortality to extreme heat. In Arizona, United States (US), consumer advocacy groups have argued that prosecutors could press charges of second-degree murder against large oil corporations for contributing towards heat-related deaths in July 2023, a heatwave that scientists claimed would have been virtually impossible without the climate crisis triggered by burning of fossil fuels.

As heat grows in frequency and intensity it merits sustained attention from geographers. This review brings together critical geographical perspectives on heat. Geographers have analysed heat in different ways, including as energy or a byproduct of industry (e.g., Harrison and Popke 2013; Gonzalez Monserrate 2023). Here though we are primarily interested in the uneven exposure of bodies (broadly understood) to heat, focusing on structural, political, economic, cultural, ecological, and infrastructural factors that shape exposure.

Our review builds on efforts to expand discourses around thermal dynamics beyond meteorological aspects—founded on western system of knowledge (Carey 2012; Wright and Tofa 2021). Such objective approaches can mask the social and political power relations associated, that are simultaneously “a means of administering and regulating life” (Starosielski 2021, 14). Instead, we contribute to ongoing multidisciplinary efforts to develop a critical temperature studies that foregrounds the material, political, cultural, and representational aspects of heat. These efforts centre lived experience—aspects of heat that are “cultural, embodied, place-based and political” (Hamstead 2024, 531)—as well as recognizing heat as a socio-technical and structural concern, acknowledging how “global heating is not a direct function of the environment but one articulated by circumstance” (Parsons 2021, 6) that is actively produced by human and non-human activities (Oppermann and Walker 2019). In doing so we argue that heat is an important and distinctive entry point from which to understand the world.

We place greater emphasis on conceptualizing extreme temperatures—beyond the physiological or societal norm, or what is healthy and tolerable. That is not to discount the importance of background, everyday or mundane experiences, which are touched on in the review. We also concentrate on warm temperatures, rather than cold. There are a wide range of spatial perspectives on energy justice implications of cold homes, which it is impossible to reflect here (e.g., Reames 2016; Bouzarovski and Simcock 2017; Waitt and Harrada 2019; Robinson 2022). However, it is challenging (and often counterproductive) to distinguish between the two given the continuum of temperature (Hernández 2015).

The paper is structured as follows. We first consider why heat has not emerged as a distinct sub-discipline in critical (primarily human) geography. We reflect on how this is complicated by the material, and at times paradoxical, qualities of heat that result in a tendency for it to be subsumed by other research agendas. We then review geographical perspectives that have attended to heat focusing on: inequalities and vulnerabilities; governance and violence; infrastructure and labor; cultures and practices; and atmospheres and elements. The paper concludes by identifying potential avenues for a sustained research agenda on critical geographical perspectives on heat.

## (Geography) can’t stand the heat

It is first useful to consider why a distinctive sub-discipline has not emerged around heat. The materiality of heat makes it somewhat tricky to analyze. Heat is often described as silent or invisible, attributed to perceptions of its ubiquity, universality, and timelessness (Beregow 2018; Khandekar et al. 2023; Singh 2023). Heat is everywhere, in both conscious and inert bodies, and traceable from the micro-scale properties of specific materials to global-scale atmospheric systems. Heat can be conceptualized as an “inescapable cosmological force,” a fine-grain form of “elemental intensities, ‘beings’ in themselves, always moving through bodies, objects, and things, perpetually affecting and producing effects” (McHugh and Kitson 2018, 157).

Thermal objects are not stable but are often highly transitory and volatile (Beregow 2018). Temperature is not simply a property of a particular entity (i.e., place, building, or body), rather it describes an “exchange of heat, a process is which everything participates” (Starosielski 2021, 2). Here, relations between bodies, and even sensory perceptions, become important. Concerning thermodynamics, Cederlöf (2024) describes a “continuous, relational mode of materiality.” Subsequently the materiality of heat is highly contextual, shaped by the built environment, local climate, access to cooling technologies, and social relations, to name but a few. Heat then is a “pervasive and immersive phenomenon that intermingles the natural and social” (Oppermann et al. 2020, 284).

The materiality of heat is closely interrelated with other aspects of what we might refer to as our immediate, ambient environment—the overlapping and shifting material forms that constitute a person's surroundings—including air flow, pollution, and humidity (Robinson and Williams 2024). For example, experience of heat is closely enmeshed with humidity as bodies feel warmer in humid conditions at the same temperature (Dunne et al. 2013). Meanwhile, in mega-cities where high toxin levels enhance respiratory problems and raise temperatures, air pollution enhances heat-related mortality (Adey 2013). Relatedly, compared to other climate-related hazards, including flooding or sea level rises, heat has often been viewed as more complex to model, owing in part to a tendency to see heat as relatively subjective (Kunz-Plapp et al. 2016; Parsons 2021).

Heat is also somewhat paradoxical (Cook and Kerr 2024). It is often presented as relatively mundane, when compared with other more dramatic weather-related hazards (Strengers and Maller 2017). Heat typically comes to our attention when acting as a stressor, for example, as negative impacts for a person's health and wellbeing, or other non-human bodies. In their account of rhythms associated with laboring in heat, Oppermann et al. (2024) recognize temperatures at which bodies no longer function as they should—a “dysrythmic” impasse. Meanwhile, critical thermal limits of life have developed over time for different species (Bennett et al. 2021). Yet, Kothari and Arnall (2019) argue that the everyday, more mundane, experiences are also central to understanding how environmental change occurs. Heat is not only acute—it can also be chronic (Bolitho and Miller 2017; Harlan et al. 2019). Furthermore, heat is not just violent, but can also sustain and energize (Hitchings 2011). Cook and Kerr (2024) describe the pharmacological quality of air, as both “poison” and “cure,” and that same framing might also extend to heat.

As such, given the complex materiality of overheating, it is challenging to isolate an analytically distinctive heat geography, or to identify at what point it becomes valuable to recognize heat as an object of attention. How then do we articulate a geographical perspective on such a tricky concept to pin down?

One common approach has been to evaluate heat as part of other discrete sectoral foci (Parsons 2021). Geographers have attended to heat as one aspect of urban climate justice (Bulkeley et al. 2013), air quality (Feron et al. 2023), energy (Cederlöf 2024), or health (Campbell et al. 2018). Heat-related challenges have formed part of a wider political ecology of air, weaving together inequalities associated with urban heat islands and heatwaves, with wider urban crises of air pollution and the manipulation of indoor environments (Graham 2015; Bouazorvski and Robinson 2022). Meanwhile thermo-dynamics are integral to understanding the materiality of energy (geographies) (Cederlöf 2024). However, subsuming heat within other domains has several drawbacks. Whilst it is important to appreciate heat in wider climate adaptation agendas, its governance often draws on practices from other domains. It is common to apply frameworks from flood management to respond to heatwave events, with limited success (Hamstead 2024). There is a risk then that the properties of heat are not fully understood.

Where a geographical understanding of heat is emerging—as we will see shortly—it has also been characterized by theoretical plurality. Plurality is something to be celebrated. Indeed, heat necessitates multi-scalar frameworks ranging from understanding of the structural, socio-ecological systems that drive heat-related inequalities, to the cultural practices and embodied experiences of heat (Mazzone et al. 2023a). We now turn our attention to critical geographical perspectives that have emerged to date.

## Critical geographical perspectives on heat

Here we consider a range of increasingly critical perspectives taken to understanding heat in geography and the wider social sciences:

1. heat inequalities and vulnerabilities;
2. thermal governance and violence;
3. conditioning infrastructures and labor;
4. warmth cultures and practices;
5. thermogenic atmospheres and attunements.

In doing so, we evaluate heat exchanges at increasingly granular scales, from relatively macro-level systemic factors, through to micro-scale impacts on individuals and bodies. There is also a large body of research in climate science and health examining meteorological and mortality concerns (e.g. Lo et al. 2023), but it would be impossible to do that justice here. Instead, we reflect on the benefits of greater integration between these multidisciplinary research agendas.

### Heat inequalities and vulnerabilities

Core to our understanding of heat is a conviction that humans, and indeed more-than-humans, can secure thermal environments that enable them to stay healthy and flourish, engendering thermal autonomy and security (Willand et al. 2021; Oppermann et al. 2024). High temperatures have been shown to have negative impacts on population health, including increasing mortality and morbidity (Jenkins et al. 2022; Mitchell et al. 2024). Wider “multi-stressors” are also associated with an inability to effectively manage thermal environments, including wellbeing, finances, mobility, social relations, and access to services (Bolitho and Miller 2017).

These stressors are unevenly distributed across bodies. Recognizing and addressing inequalities and vulnerabilities associated with heat extremes is therefore integral to achieving climate justice (Schlosberg and Collins 2014; Sultana 2022). However, media representations of heat tend to bind people in a shared perception of their surroundings, rather than making unevenness visible (Starosielski 2021). Some populations are disproportionately vulnerable, whether physiologically (e.g., age, health), or across other intersectional axes of inequality (e.g., race, income, class, gender, disability) that mean they are less able to adapt, shelter, care, or labor in the face of extreme heat (Dunne et al. 2013; Mashhoodi 2021; Mikulewicz et al. 2023; Chakraborty 2024). For the more-than-human, certain species or environments have a heightened vulnerability to temperature change, for example, coral bleaching events during prolonged heat stress (Westgate 2023). It becomes important to acknowledge the specificities of local context and communities, and how what might be considered essential to ensure thermal security in one context, may not be in another (Walker 2022).

Evidence of inequalities and vulnerabilities is wide-ranging, often quantitatively mapping socio-spatial vulnerability patterns (Emrich and Cutter 2011; Lindley et al. 2011; Weber et al. 2015; Mitchell and Chakraborty 2018; Navarro-Estupiñan et al. 2020; Robinson et al. 2025). The spatial unevenness of urban heat islands, in particular, have been emphasized (Sarricolea et al. 2022). Jung et al. (2024) analyses heat risk, urban form, and social vulnerability across Belo Horizonte Metropolitan Region, Brazil, showing how non-White, low income, and older residents are disproportionately exposed. In Detroit, US, low-income minority communities have increased vulnerability, partly because of higher pavement density in their neighborhoods (Sanchez and Reames 2019). In Montreal, Canada, whilst 0.7% of the population identify as Indigenous, they represent 12% of the homeless population, making them disproportionately vulnerable (Fan and Sengupta 2022). In New York City, US, high call volumes about power outages are increasingly common during heat waves, especially for low income, racial minorities, that have high energy burdens (Marcotullio et al. 2023). Colucci et al. (2021) evidence thermal inequalities in carceral environments, where complex social and infrastructural factors enhance vulnerability, including a reliance on factors beyond their control to create a healthy living environment.

At the other end of the spectrum, relatively privileged communities increasingly protect themselves from the heat in artificially controlled microclimates (Graham 2015; Marvin and Rutherford 2018). Air conditioning is often expensive, energy-intensive, and requires a stable electricity connection (Singh 2023). Globally, air conditioning use is concentrated amongst high-income households (Davis et al. 2021; Romitti et al. 2022). Not only does this manifest in inequalities between households in ability to cool, but typically affluent, air-conditioned enclaves transfer heat to other communities, via heat exchangers that dump heat at the front of buildings (Graham 2015; Yip et al. 2020). Examining air-conditioning practices in Wuhan, China, Courtney (2023, 13) identifies how “the hotter their city gets the more they need to cool their homes, the more they try to cool their homes the hotter their city becomes.”

As climate change intensifies, the geographies of where air conditioning is required are being reconfigured, and extreme heat may shift choices about where people call home (Hitchings 2011). A recent assessment of the future cooling gap in megacities across the Global South estimate that between 33% and 86% of the population in Sub-Saharan African and South Asia are likely to be unable to access cooling infrastructures whilst experiencing growing heat stress by the middle of the century (Mastrucci et al. 2019; Mastrucci et al. 2022).

In response, multiple conceptual frameworks for understanding locally specific inequality related to cooling have emerged, including thermal inequality (Mitchell and Chakraborty 2018; Parsons 2021; Mashoodi and Kasraian 2024), systematic cooling poverty (Zhang et al. 2023; Mazzone et al. 2024), thermal inclusion (Oppermann et al. 2024), thermal insecurity (Hamstead 2024), or an extension of energy poverty concepts classically applied to colder temperatures (Hernández 2016; Thomson at al. 2020; Tabata and Tsai 2020; Yip et al. 2020). Systematic cooling poverty has been defined as:

encompass[ing] intricate layers of physical, social and intangible infrastructural deficiencies, impeding the provision of essential services necessary to ensure thermal safety during extreme heat episodes. (Mazzone et al. 2023a, 1026)

Hamstead (2024) uses thermal (in)security to understand the threats associated with heat and cold, intertwined with housing, energy and related social determinants of health. These concepts and frameworks share a common concern—that insecurity or vulnerability of communities arising from extreme heat is a structural form of inequality and violence.

### Thermal violence and governance

Like many forms of violence, the violence of heat takes multiple forms, ranging from the overt deadly consequence of extreme temperatures to the hardly recognizable, mundane everyday realities of heat (Springer and Le Billon 2016). Indeed, extreme temperatures have been used to directly administer violence, as exemplified in the “sweatboxes” of the American South, a form of solitary confinement in hot conditions used to enact racialized violence in plantations and prisons (Starosielski 2018). Thermal violence also extends to the wider socio-economic and political structures that reflect an uneven distribution of power to manipulate and mediate heat—what Starosielski (2021) terms “thermopower.”

A structural understanding of exposure of marginalized populations to heat acknowledges that the agency of individuals to respond and cope is often limited (Abbott et al. 2015; Jamei et al. 2016). Removal of thermal autonomy in a way that restricts ability to respond to heat—whether deliberately or accidentally—is a form of violence (Starosielski 2018). For example, in the case of brick production in South Asia, labor arrangements mean many workers are debt-bonded to the kilns, forced to continue to work in extreme conditions until they are repaid (Parsons et al. 2024).

Heat governance is also highly relational, dependent on often hierarchical relationships between a multitude of actors (e.g., friends and family, landlords, policymakers) and systems (e.g., housing rights, tenure, welfare support, utility provision) that shape the thermal (in)security of individuals (Robertson et al. 2024). Given the growing tendency towards privatization of urban services upon which people rely to fulfil basic needs—including access to water or electricity networks for cooling—there has been an ongoing restructuring of who has agency over the built environment and heat stress mitigation (Petrova and Prodromidou 2019; Anwar et al. 2020).

These relations are predicated on firmly embedded capitalist and colonial structures. Heat-related inequalities are:

felt, materially, “on the ground” by everyday people because of already experienced uneven development and the relational production of the anthroposphere by capitalism. (Colucci et al. 2023, 226)

Not only does this facilitate the individualizing of responsibility for cooling amongst select groups (Bosca 2023), but political structures also enable control and manipulation of atmospheres and thermal systems (Beregow 2018; Furuhata 2022). In Singapore, air-conditioning infrastructure has become central to life for most citizens, part of a wider project of nation-building (McNeill 2019). Marks and Connell (2023) show how thermal governance is highly unjust in Bangkok, Thailand, as actors that profit from heat, or actively contribute towards it, including real estate developers and automobile companies, are typically shielded from its effects. In the case of Nigeria, Ajibade and McBean (2014) show how weak housing rights mean that the urban poor are forced to encroach further into landscapes that engender heat stress, and to adopt environmentally intolerable coping strategies. Austerity urbanism and unequal investment across cities has also amplified heat inequalities in recent years (Zuniga-Teran et al. 2021).

Deep colonial and segregation legacies can be traced in historical planning decisions that shape heat exposure (Kobi 2023; Macktoom et al. 2023; Yee and Kaplan 2022). Historical redlining in US cities, based on discriminatory and racist housing policies, play a role in disparities in heat-related illness in the present day (Wilson 2020; Li et al. 2022; Manware et al. 2022). Evidence of individual and collective vulnerability as a product of historical, colonial dynamics can be seen in Yarrabah, Australia, where the attributable impacts of climate change are inextricably linked to the state-sanctioned expansion of resource frontiers into Aboriginal lands (Jackson 2023). Here, the erosion of livelihoods, language, knowledges, sense of place and sovereignty because of massacre and displacement are interconnected with climate-related changes in seasons, which also impacts on Indigenous knowledges.

To date, urban climate governance processes have often lacked recognition of perspectives of diverse communities at the sharp end of heat stress (Yazar and York 2022). Jonsson and Lundgren (2015) evaluate how municipal planners in Sweden construct understanding of heat vulnerabilities without considering the extensive local knowledge of carers, and vulnerable communities themselves (see also Anwar et al. 2022). In Ahmedabad, India, policies to address urban heat have been translated between cities without accounting for local nuances in different places (Khandekar et al. 2023).

A lack of understanding of community perspectives in heat governance is especially prevalent in informal settlements. Ethnography in urban kampongs in Jakarta, Indonesia, records how residents collaboratively build vernacular, communal cooling infrastructures using limited resources (Salsabila et al. 2023). Despite successfully cooling residents and building social relationships, government officials fail to recognize their importance in heat mitigation—symbolic of a wider failure to acknowledge the needs of informal communities where development is considered “unplanned” or erratic by the state (Chu and Michael 2019; Michael et al. 2019; Salsabila et al. 2023).

How heat is problematized in governance matters. In Australia, heat policy has failed to recognize both the role of humidity in amplifying chronic effects of heat (Bolitho and Miller 2017; Oppermann et al. 2017). In the case of relatively mountainous Nepal, a focus on other climatic hazards such as floods, landslides, and earthquakes mean that the dangers of extreme heat for urbanites living on the humid plains of the Terai region remain invisible in governance, despite planning failures enhancing their exposure (Kandel and Shyangtan 2023). Understanding of structural inequalities and violence rarely shapes thermal governance, if considered at all. In a review of over 7,500 local climate action plans globally, urban heat is systematically excluded in places where local climate and population dynamic make it appropriate (Ulpiani et al. 2024).

Heat is also generating new forms of climate governance and urbanism. In post-industrial Buffalo, US, the city has sought to counteract industrial decline by branding itself a place for climate migrants to seek cool—a climate haven of sorts (Hamstead 2024). Here, lower temperatures, relative water and energy security, and cheap real estate suggests an increased capacity to absorb a growing population because of future climate-related migration. However, new forms of climate urbanism do not always align with the experiences of thermal insecurity amongst existing communities. Indeed, Thomas (2024) argues that adaptation has become an opportunity for the accumulation of capital, with political and economic elites profiting from adaptation efforts.

### Conditioning infrastructures and labor

Heterogeneous materials and infrastructures are also involved in the thermal management of bodies, both directly and indirectly. Infrastructures—whether through failure, inefficiency, affordability or inaccessibility—play a key role in exposure of populations during heatwaves, especially for the most precarious (Petrova and Prodromidou 2019; Mazzone et al. 2023b; Yáñez Serrano et al. 2024). Infrastructural failure is most pertinent for systems that are slow to change, outpaced by climatic change for example, including buildings that are no longer fit for purpose several decades after their design. Inaccessibility is particularly acute in places that are “off-grid” and unable to benefit from access to networks that could mitigate temperature extremes (Anilkumar et al. 2022), or where these networks are unreliable (Longden et al. 2022).

Identification of a range of cooling “services”—electricity, water, sanitation, shelter, vegetation, and shade—highlights the importance of interrelations between heat and other socio-technical infrastructures and systems (Opperman et al. 2024). The built environment is especially integral to achieving climate justice (Klinsky and Mavrogianni 2020). Material changes to buildings are made in response to heat stress, for example, installing shutters, loft insulation, or air conditioning (Thomson et al. 2019). A wider range of infrastructures also intersect to shield bodies from extreme temperatures, from the physical (green space, water supply, air conditioning, electricity supply, buildings) to the social (networks, knowledge of heat, literacy) (Zhao et al. 2018; Mazzone et al. 2023b). For example, in Hyderabad, India, a lack of access to sanitation facilities has been shown to prevent women and girls from taking in sufficient fluids to mitigate excessive heat (Sahu et al. 2013; Anilkumar et al. 2022). These diverse infrastructures are part of a complex assemblage of relations that shape heat exchanges, drawing together humans, non-humans, and objects into collective forms (Cook and Kerr 2024).

One of the most important infrastructural aspects of heat mitigation is the fabric of homes. Nowhere is this more apparent than informal settlements. Remote sensing of surface temperatures in Ahmedabad, India, illustrate how informal settlements are particularly exposed to locally high temperatures, especially larger settlements (Wang et al. 2019). An often-defining feature of informal housing is a lack of permanence, as well as an inability to protect against extreme climatic conditions. Poor quality, cheap building materials, such as plastic sheeting and corrugated metal, foster chronic heat conditions (Mahabir et al. 2016; Anilkumar et al. 2022). Not only does building fabric enhance local temperature, but informal settlements are often located in places that already experience high temperatures (Mehrotra et al. 2018; Wang et al. 2019; Pasquini et al. 2020; Damte et al. 2023; Obe et al. 2023). Resultant, localized heat extremes are often underestimated by weather stations, as in humid Makassar, Indonesia (Ramsay et al. 2021).

Urban density also impedes on air movement, enhancing heat-related mortality (Pramanik et al. 2022). In informal settlements, closely situated buildings and low levels of vegetation prevent the circulation of air, a crucial part of cooling spaces (Van de Walle et al. 2022; Salsabila et al. 2023). A poverty of space has been shown to enhance vulnerability to extreme heat, including amongst those living in small flats in Hong Kong (Song et al. 2020; Yip et al. 2020; Lo et al. 2022). Dense urban form and precarious housing conditions were central to mortality during a 2003 heatwave in France, which killed more than 15,000 people (Keller 2013). There is a distinctively sensory experience to urban density, reflected in intensifying humid heat (Chen and McFarlane 2023). Unevenness in urban density, and relatedly shade, has been linked to colonial legacies in Karachi, Pakistan, where a distinct “Black Town” was constructed, characterized by densely packed streets and limited green space, compared to the sparse neighboring “White Town” for European residents (Hamstead 2024).

Heat is also deeply embedded in architectural and urban transformation. In Chongqing, China, staying cool has shifted as socialist era buildings from the 1960s are replaced by high-rises, enabled by the widespread electrification of the city. Subsequently many residents have transitioned from communal cooling to private, air-conditioned spaces (Kobi 2023). Historically in the Brazilian Amazon, vernacular knowledge played a central role in passively cooling dwellings, however, this has been negated by a hybridization of modern materials (e.g., cement, corrugated metal) which have in fact nullified natural cooling systems (Mazzone 2020). Amongst carceral populations, construction materials are overwhelmingly concrete, brick, or metal—effective conductors of heat—and prison design means that there is typically little access to shade and heat stress is common. Architecture therefore acts in a way that is punitive, perpetuating state violence (Colucci et al. 2023).

Technological infrastructures also allow humans to “manufacture their own air” (Graham 2015, 192), nullifying temperature fluctuations. In warmer cities, the middle class and wealthy increasingly expect continuous access to cool manufactured air, resulting in energy-intensive, unsustainable transformations of urban living (Hitchings 2011; Winter 2013; Walker et al. 2014; Graham 2015; Osunmuyiwa et al. 2020). A rise in air-conditioning has led to two distinct phases of modernity—preconditioned and conditioned (Winter 2013; Chang and Winter 2015). Yet different zones of thermal privilege and violence endure (Starosielski 2021). Despite widespread use of air-conditioning in Hong Kong, energy and cooling is not seen as a right, and access to air conditioning is shaped by complex financial and cultural characteristics (Fuller et al. 2019). In the favelas of Rio Di Janeiro, Brazil, energy inefficient air conditioning infrastructures motivate illegal electricity connections, in turn driving blackouts when temperatures are high (Mazzone 2020; see also Klininberg 2015). Climate control also enable conservation practices to evolve, as evidenced in the case of London's Kew Gardens, UK, where new volumetric enclosures has allowed for the movement of plants between different contexts via the construction of “enclosed conditions decoupled from local climate … in some cases the last remaining sites, of these natures” (Rutherford and Marvin 2024).

Vegetation and greenspace play an important role in cooling, especially in cities where urban heat island effects are acute (Zhao et al. 2018; Xu et al. 2022). However, its cooling potential is spatially and temporally uneven (Venter et al. 2020; Yao et al. 2020; Wu et al. 2023; Chen 2024). For example, in built-up areas of tropical cities, including Kampala, Uganda, vegetation has a shorter growing season compared to relatively rural areas, owing to intense land surface temperatures, thus reducing the cooling benefits associated (Kabano et al. 2021; Zhang et al. 2023). Climate refuges, public spaces in cities that provide protection from climatic extremes (e.g., public transport, parks, green spaces, libraries) are also increasingly critical, everyday infrastructures (Amorim-Maia et al. 2023; Braun et al. 2024). However, they are embedded in existing urban landscapes and historical power relations. Amorim-Maia et al. (2023) analyze 200 designated climate shelters in Barcelona, Spain, finding they rarely address intersecting vulnerabilities of marginalized populations.

Infrastructural labor—the social capacity to repair and care—also shapes exposure to extreme heat (Alam and Houston 2020; Stokes and De Coss-Corzo 2023). Care as infrastructure is under-acknowledged in the capitalist systems, given how deeply embedded it is in everyday life, and its social, as much as economic, value. Yet care and repair enable community adaptation to heat, for example, when caring for vulnerable people or during a heatwave (Carr 2023). Community members in the US describe feelings of responsibility for the thermal security of pets, children, and older family members (Hamstead 2023). Much of the labor of climate adaptation, including to temperature extremities, is free and invisible, a key climate justice challenge (Johnson et al. 2023). However, Anilkumar et al. (2022, 50) also emphasize how, in Indian cities, the proliferation of cooling technologies has led to the “emergence of a rich culture and economy of maintenance and repair work.”

Finally, there is a deep concern about the ways in which “productive” labor exposes people to extreme heat (Kleinheisterkamp-González 2023). Productive labor decreases under heat stress (Johnson et al. 2023), projected to decline by up to 20% in hot months by the middle of the century (Zander et al. 2015). Heat-related illnesses can lead to a loss of wages, or new medical bills for people (Sett and Sahu 2014). Despite calls for a right to shade, especially where labor relations mean precarious workers are required to be outdoors at the hottest part of the day, shade is rarely regarded as a civic or common resource. Instead, it is highly bureaucratic and securitized (Macktoom et al. 2023). Yet these dynamics are not always linear, as in the case of agricultural workers in Cambodia, who have left the sector due to climate-related precarity to subsequently experience heat stress as brick builders (Parsons 2021). Singh and Basu (2020) record experiences of migrant and non-migrant families in Karnataka, India, where heat is making farming less profitable, and more laborious. Here, commuting and migration can be seen to alleviate vulnerability for some family members whilst exacerbating it for those who spend most of the time at home. This illustrates how wider cultures, practices, behaviors, and norms also play an important role in thermal dynamics.

### Warmth cultures and practices

For Hulme (2015, 1), “climate—as it is imagined and acted upon—needs to be understood, first and foremost, culturally.” Rising temperatures are causing, especially vulnerable, people to adapt their conditions to remain cool (Serrano et al. 2023). Behavioral practices for thermal management vary hugely depending on local context, from actively ensuring adequate nutrition and water, to reducing work or changing work patterns, moving or migrating to cooler spaces, adjusting clothing, showering more often, encouraging ventilation, and directly space cooling.

Practices are socially constructed and highly uneven, underpinned by geographically embedded cultural differences, routines, habits, perceptions, norms, memories, and experiences, each of which shapes exposure to thermal stimuli. Robertson et al. (2024, 3) highlight how for some households “everyday activities are merely slowed or altered until a heatwave passes,” whilst for others they are temporally dissonant prompting a crisis or rupture that can further enhance vulnerability.

Perceptions of comfort vary depending on local climate, and how people have historically dealt with extreme temperatures (Wilhite 2013). Through engagement with the cooling practices of international students in Melbourne, Australia, Strengers and Maller (2017, 1433) redefine the goal of adaptation “as achieving tolerable, interesting, manageable, exciting, challenging and curious conditions rather than pursuing [solely] comfort, familiarity and safety.” Notions of what it means for a society to be “civilized,” reflected in retaining formal work attire, or gendered norms of masculinity, including working outside irrespective of the temperature, are also reflected in coping mechanisms (Hitchings 2011).

Oppermann and Walker (2019) argue that rather than seeing heat as one element of practice, it is better understood as a dynamic form of energy in which all practices are immersed. The body is not simply a passive recipient of temperature, but rather an “active agent of adaptation, self-regulation and transformation” (Mazzone and Khosla 2021, 1). Although often mundane, practices provide a mechanism for household resistance and disobedience in response to wider structural forces that limit thermal agency, as evidenced in relation to austerity (Petrova and Prodromidou 2019). Notably though, people's capacities to adapt to heat and shift practices are “strikingly uneven,” often constrained by finances, mobility, and socio-economic status (Thomson et al. 2019).

Practices of adaptation are strongly rooted in local and historical context. Oppermann and Walker (2019) show how, in northern Australia, colonial conquest translocated European practices of coping with heat from temperate to tropical regions, including work hours and the use of protective clothing. Whilst heat is a product of existing cultural formations and practices, they evolve over time, in turn producing new practices and cultures (Courtney 2023; Yao 2023). In Wuhan, China, a city well-known for its heat, everyday practices of resisting heat have shifted from Maoist era (1949–1976) when outdoor bamboo beds were common, to doors being closed to neighbors as air conditioning has been introduced (Courtney 2023). In turn, these socio-economic and technical changes have eroded community cohesion that comes from spending time outdoors with others (Hitchings 2011).

Practices and vulnerabilities also change over a life course, as people age, have children, or move house, learning to manage new environments and sensitivities in the process (Royston 2014; O'Sullivan and Chisholm 2020). Through oral histories in South Australia, older residents explain how they have become increasingly reliant on cooling technologies over their lifetime (Goodchild et al. 2020). These patterns have differential local impacts, for example, enhancing vulnerability where populations are ageing (Wang et al. 2023; Carr et al. 2024; Falchetta et al. 2024).

There has also been a distinctive focus on problematizing air-conditioning practices. Transnational transitions are occurring in air-conditioning use, through a wide range of shifting multi-sited practices, from the mechanical cooling of offices to homes (Shove et al. 2014). Air-conditioning masks constant and evolving adaptations to weather that would otherwise be made (Strengers and Maller 2017). In Japan, high levels of affluence and relatively cheap technologies mean that air-conditioning is deeply embedded, amplified by a cultural tendency towards formality with office workers retreating to air-conditioned spaces to retain work attire (Hitchings 2011). Transitions are co-producing new social constructions of what it means to be comfortable whilst eroding local knowledge about how to stay cool, with this knowledge increasingly relegated to technical experts (Wilhite 2013). However, air-conditioning is also spoken of “lovingly... as a constant companion in their lives” by young people in Qatar, a desert climate where the technology has been widely adopted (Hitchings 2020, 120). Culture and practice provide new perspectives on mitigating heat, but this can be extended to emphasize embodied and situated experiences of heat, though consideration of atmospheres and attunements (Verlie 2019).

### Thermogenic atmospheres and attunements

Heat is something that cannot simply be represented with a thermometer, but is also lived, both individually and communally. Hepach and Hartz (2023, 211) advocate for a phenomenological approach to experiences of rising temperatures, showing how “heatwaves [also] signal an existential loss: not just the loss of something, but the turning unfamiliar of one's world.”

Whilst emphasizing the importance of the cultural aspects of heat, the concept of atmospheres has been used to acknowledge a “persistent material or meteorological presence, whether real or imagined, which envelops or unsettles the human subject” (Gandy 2017, 355; Tripathy and McFarlane 2022). Emphasis has been placed on affective atmospheres, that foreground relational, embodied and sensory experiences, for example, that of air or light, as well as the wider political and cultural connotations associated (Anderson 2009; Gandy 2017). For Gandy (2017), it is the “criss-crossing” of these sensations that constitute urban atmospheres, and everyday life in cities, including thermal atmospheres.

A crucial part of how people understand the world is through weather, with bodies understood as co-produced by, through, and with, the weather (Ingold 2010; Neimanis and Walker 2014; Morris and Endfield 2016). Affective atmospheres reflect how community experiences of heat are entangled with feelings about—or memories of—where they live. Here, extremities and variabilities in temperatures become active participants in place-making (Adams-Hutcheson 2019; Endfield 2019).

Affective atmospheres of heat are apparent amongst white settler populations in Western Australia, who balance grief about extreme heat with hope, as part of a process of “relational placemaking” that helps them to come to terms with the ongoing apocalypse (Tschakert et al. 2024). Examining farming practices in the troposphere, Adams-Hutcheson (2019, 1004) evaluates the vibrant materialities of weather, highlighting the “relational weight of atmospheres” and how the intense heat of summer “encapsulates human and non-human bodies, moods and actions.” Attention to affective atmospheres can also disrupt meteorological understandings of temperature. In New Zealand, farmers climate perceptions have been found to be inconsistent with historical temperature records (Niles and Mueller 2016), emphasizing the need to consider embodied, situated, and relational experiences of heat.

Embodied experiences of heat are also historically situated (Endfield 2016; Frazier 2019). In Hawai’i, an account of the commoditization of ice, an idea that only developed as part of American imperial power and indigenous dispossession in the mid-1800s, provides a sense of the sensory thermal experiences of ongoing settler colonialism, and how people's perception of temperature in everyday life shifts under colonization (Hobart 2023). Once termed “India's naturally air-conditioned city,” discussion of warming weather in Bengaluru has been used to capture wide ranging embodied experience of the transformation of the urban environment in a way that is increasingly overwhelming for its occupants. Anchored in class-specific and historical relationships with the city, “critiques about rising temperatures are also critiques of rapid urban growth” (Frazier 2019, 442).

One way of attuning to lived, bodily, and sensory experiences of heat has been through a focus on rhythm. Through thermal rhythmanalysis, Oppermann et al. (2020, 275) invite us to attune to heat as an energetic and lively form, with its own agency. In this way, rhythms that are perceived as external to the body become known, coalescing into polyrhythmic assemblages as “energetic thermal flows are variously exchanged, accumulated and dispersed within and around human bodies.” For example, farmers have been shown to attune to the rhythms of livestock and crops, based on extensive knowledge of weather, place, and seasons (Adams-Hutcheson 2020). Furthermore, rhythmic analyses also emphasize the importance of temporal relations. In evaluating social practices of coping with and adapting to heat in homes, it is important to emphasis not only short-term but also longer-term responses to heat extremes (Robertson et al. 2024).

Breathing can also act as a way of attuning to—or “witnessing”—heat (Verlie and Neimanis 2023). For Verlie and Neimanis (2023, 118), “to breathe climate catastrophe is to witness one's own body-in-and-as-part-of-the-climate.” However, it is also acknowledged that nobody can witness the entire climate catastrophe through just their own body. Accordingly, geographers have argued for attuning to the “more-than-individual” and “more-than-human” experiences to fully understand climate-related heat (Verlie and Neimanis 2023). Rooted in Indigenous and feminist theories, more-than-human perspectives have grown in the face of climatic change, recognizing how human suffering is dynamically entangled with the suffering of non-human others (Bennett 2020; Celermajer et al. 2022; Tschakert 2022; Verlie 2022). Indigenous perspectives also disrupt Western accounts of weather and climate built on a strict division between people and their environment, instead emphasizing that they “co-constitute both people and place in embedded, relational, more-than human ways” (Wright and Tofa 2021, 2). More-than-human ontologies can be used to emphasize how thermal dynamics transgresses the distinction between human and non-human (Oppermann and Walker 2019).

## Towards a critical heat geographies

Heat—especially heat stress and extremes—is worthy of sustained attention from geographers, as it grows in its frequency, intensity, and urgency in the coming decades (Kaika et al. 2023). In this review, we brought together diverse, often disparate, geographical perspectives on heat. In doing so, we have highlighted the uneven exposures of diverse bodies to heat. We have shown how a range of structural drivers shape exposure to high temperatures, from people living in insecure housing in informal settlements where building materials and a lack of rights endangers health, to air-conditioned microclimates that enable a variety of species to live in environments that would otherwise be inhospitable. Yet we have also emphasized the importance of the lived, phenomenological experiences of individuals and communities exposed to thermal extremes. Each perspective shows it is insufficient to only measure temperature meteorologically.

Materiality complicates the study of heat, simultaneously conceptualized as silent, immersive, pervasive, violent, multi-scalar, volatile, extreme, and mundane (Beregow 2018; Khandekar et al. 2023; Singh 2023). However, arguably critical human geographers are uniquely positioned to analyze these thermal qualities that are all-encompassing—to capture a phenomenon that is at once meteorological, social, structural, political, material, more-than-human, sensory, and subjective. Whilst the pervasiveness of heat, and the impossibility of defining boundaries between distinctive thermal objects, makes it challenging to delineate a specific field of study, it is important to foreground heat (Oppermann et al. 2020).

The idea of a continuum is used in energy geographies scholarship to reflect the spectrum of dynamics from both energy production and consumption that give rise to vulnerability (Hernández 2015; Jenkins et al. 2020; Bouzarovski 2022). Here, there is a similar continuum of socially-mediated heat exchanges, along which a diverse range of bodies, infrastructures, fuels, climates, and more exist. Heat then can be a novel entry point via which to study the world. Thermal dynamics are integral to multiple sub-disciplines, from energy geographies to hazard geographies, in turn necessitating multi- and inter-disciplinary perspectives that enable us to fully comprehend the diversity of relations along this continuum.

What then might be important avenues of research to be considered as part of critical geographical perspectives heat? One key aspect, highlighted by Campbell et al. (2018), is the need for further sustained evidence from diverse contexts across the Global South where exposure to heat stress is often most acute and uneven, yet detailed qualitative accounts of the lived and embodied aspects of overheating are currently underrepresented. Beyond this, by way of concluding, we identify three potential avenues, each framed as binaries that future research might seek to overcome. We consider hot and cold; indoor and outdoor; and physical and human. Our focus on relatively broad binaries avoids being too prescriptive and emphasizes the potential for contributions from across the variety of perspectives discussed in the section “Critical geographical perspectives on heat.”

**Hot and cold:** In this review, we primarily focus on heat, and therefore actively contribute to an analytical distinction commonly made between the experiences and stresses arising from exposure to high and low temperatures. However, empirically there is considerable benefit to understanding temperature as a continuum (Hernández 2015). There is therefore a need to better understand interrelations between the infrastructures, practices, and vulnerabilities associated with coping with extreme heat and cold concurrently (Harrison and Popke 2013; Day et al. 2016; Petrova and Prodromidou 2019; Sera et al. 2019; Thomson et al. 2019). Efforts to adapt to extremely warm temperatures may endanger adaptive capacity in colder extremes, as shown in parts of northern urban China where people turn to summer air conditioning units to deal with harsh winter cold, without the benefit of other networked heating systems that are more fit for purpose (Robinson et al. 2018). Furthermore, this would contribute to a better understanding of the cumulative effects on bodies of exposure to a multiplicity of stressors, including under and overheating, but that also extends to other ambient dimensions such as air quality and noise (Robinson and Williams 2024).

**Indoor and outdoor:** There is a tendency in heat research and governance, to privilege the outdoor at the expense of the indoor. Hamstead (2024) problematizes neglect by the state of the private spaces in which heat stress becomes important, especially homes. Calls for indoor environments to be prioritized highlight legitimate concerns about how societies that are increasingly enveloped in air-conditioned environments may in fact forget entirely what is to live in the climate outside (Hitchings 2011; Wilhite 2013). Yet these debates also risk perpetuating an artificial distinction between the indoor and outdoor (Day Biehler and Simon 2011). Instead, scholars have conceptualized the boundaries of the home as porous (Burrell 2014; Blunt and Sherringham 2019), recognizing the connection between the home and the wider environment or city, including materially via the surrounding air (Bouzarovski and Robinson 2022). For example, Burrell (2014) describes how metaphorical outside forces “spill over from the street” into the home, and vice versa. Given the near constant mobility and exchange of heat, and therefore its pervasiveness (Adam's-Hutcheson 2020; Macktoom et al. 2023), it is beneficial to breakdown the binary between the indoor and outdoor as part of a critical understanding of the geographies of heat.

**Physical and human:** Finally, there are benefits to physical geographers researching together with critical human geographers to reduce the adverse impacts of extreme temperatures and promote environmental justice (Colucci et al. 2021; Beray-Armond 2022; Engel-Di Mauro 2022). Colucci et al. (2021, 226) argue that:

a focus on the materiality of air – how air molecules absorb and disperse energy, and change the human/biophysical experience of earth's climatic systems – is a starting point for physical and social scientists to work together in examining how atmospheric and climatic patterns are both unevenly felt and influence the conditions of human lives and deaths.

From this starting point there is the potential for trans-disciplinary approaches to move beyond politically blind understandings of anthropocentric capitalism, expanding human and physical geography understandings of heat beyond a simple meteorological event to situate it within political, economic, and cultural systems, and consider the power relations associated.

Analysis of both social and physical dynamics concurrently would improve the ability to tackle structural heat-related inequalities, enrich evidence of thermal and climatic injustices, deepen meteorological understandings of temperature change, and democratize processes of producing climate knowledge (Colven and Thompson 2019; Tyszczuk 2021;Beray-Armond 2022). Greater engagement between physical geography and critical human perspectives would also enrich understandings of more-than-human and multi-species justice, for example, the uneven burdens of heat on ecological systems (Wright and Tofa 2021; Celermajer et al. 2022). Currently, perspectives from physical geography are underutilized in critical geographical research on heat, and vice versa (Colven and Thompson 2019), with notably exceptions (e.g., Lave 2015). These three avenues would enhance a critical geographical perspective on heat—an issue that will only grow in importance as thermal extremes occur with greater frequency and intensity throughout the twenty-first century.

## Notes

1. Travel on the Waterloo and City line during July 2018 and you can expect to experience a balmy 26.5 °C, still well within recommendations for comfortable ambient temperature. But travel on the deep Bakerloo line, via trains introduced in the 1970s without air conditioning, and *average* temperatures are 32.4 °C.
